# Supplementary material for: Conditional chemoconnectomics (cCCTomics) as a strategy for efficient and conditional targeting of chemical transmission
Source: eLife. 2024 Apr 30;12:RP91927. doi: 10.7554/eLife.91927 (PMC11060718; doi:10.7554/eLife.91927)
Supplement: Figure 3—source data 3. [file elife-91927-fig3-data3.zip › Figure 3 source data3 part 2.zip!/Figure 3-Source data 3-part2/1025-13-CRISPResso_Report_ack040l3.zip!/CRISPResso_on_1025_14/9.Alleles_frequency_table_around_sgRNA_agcacaaacttggcggtgac.pdf]

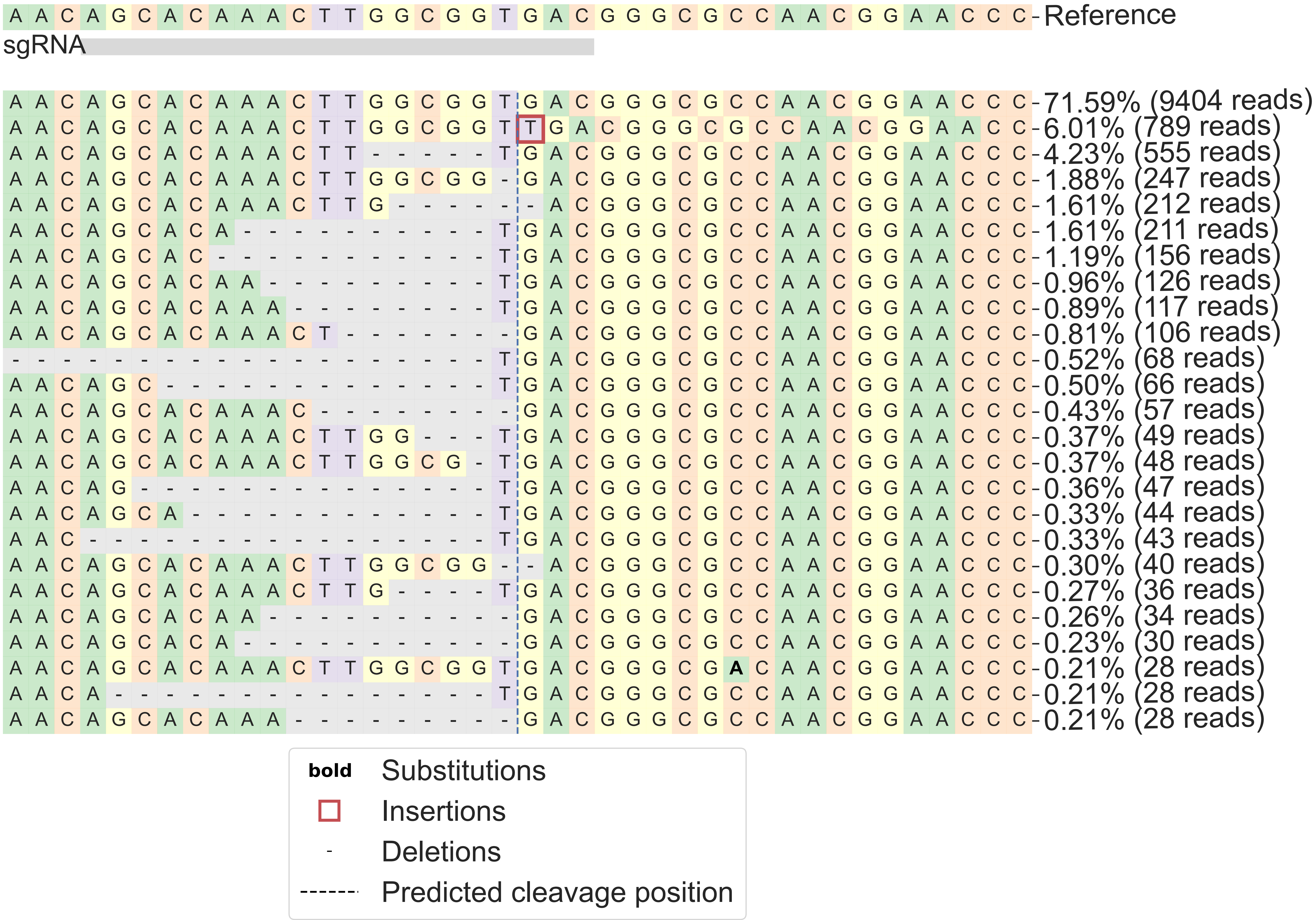
AACAGCACAAACTTGGCGGTGACGGGCGCCAACGGAACCC Reference
sgRNA
AACAGCACAAACTTGGCGGTGACGGGCGCCAACGGAACCC 71.59% (9404 reads)
AACAGCACAAACTTGGCGGTTGACGGGCGCCAACGGAACC 6.01% (789 reads)
AACAGCACAAACTT-----TGACGGGCGCCAACGGAACCC 4.23% (555 reads)
AACAGCACAAACTTGGCGG-GACGGGCGCCAACGGAACCC 1.88% (247 reads)
AACAGCACAAACTTG------ACGGGCGCCAACGGAACCC 1.61% (212 reads)
AACAGCACA----------TGACGGGCGCCAACGGAACCC 1.61% (211 reads)
AACAGCAC-----------TGACGGGCGCCAACGGAACCC 1.19% (156 reads)
AACAGCACAA---------TGACGGGCGCCAACGGAACCC 0.96% (126 reads)
AACAGCACAAA--------TGACGGGCGCCAACGGAACCC 0.89% (117 reads)
AACAGCACAAACT-------GACGGGCGCCAACGGAACCC 0.81% (106 reads)
-------------------TGACGGGCGCCAACGGAACCC 0.52% (68 reads)
AACAGC-------------TGACGGGCGCCAACGGAACCC 0.50% (66 reads)
AACAGCACAAAC--------GACGGGCGCCAACGGAACCC 0.43% (57 reads)
AACAGCACAAACTTGG---TGACGGGCGCCAACGGAACCC 0.37% (49 reads)
AACAGCACAAACTTGGCG-TGACGGGCGCCAACGGAACCC 0.37% (48 reads)
AACAG--------------TGACGGGCGCCAACGGAACCC 0.36% (47 reads)
AACAGCA------------TGACGGGCGCCAACGGAACCC 0.33% (44 reads)
AAC----------------TGACGGGCGCCAACGGAACCC 0.33% (43 reads)
AACAGCACAAACTTGGCGG--ACGGGCGCCAACGGAACCC 0.30% (40 reads)
AACAGCACAAACTTG----TGACGGGCGCCAACGGAACCC 0.27% (36 reads)
AACAGCACAA----------GACGGGCGCCAACGGAACCC 0.26% (34 reads)
AACAGCACA-----------GACGGGCGCCAACGGAACCC 0.23% (30 reads)
AACAGCACAAACTTGGCGGTGACGGGCGACAACGGAACCC 0.21% (28 reads)
AACA---------------TGACGGGCGCCAACGGAACCC 0.21% (28 reads)
AACAGCACAAA---------GACGGGCGCCAACGGAACCC 0.21% (28 reads)
bold Substitutions
Insertions
- Deletions
Predicted cleavage position
